# Down boy! A case of acute abdomen following a dog bite to the scrotum

**DOI:** 10.1186/s12887-019-1548-x

**Published:** 2019-05-28

**Authors:** Edwin S. Palmer, Phitsavanh Saysamoneyeu, Jennifer M. Siu, Annkham Thammaseng, Indi Trehan

**Affiliations:** 1 Lao Friends Hospital for Children, Luang Prabang, Lao People’s Democratic Republic; 2 Boston Children’s Hospital, Boston, MA USA; 3 Luang Prabang Provincial Hospital, Luang Prabang, Lao People’s Democratic Republic; 4 Department of Otolaryngology – Head & Neck Surgery, University of Toronto, Toronto, ON Canada

**Keywords:** Dog bite, Acute abdomen, Scrotal trauma, Perforated bowel, Case report

## Abstract

**Background:**

Dog bite injuries are an ongoing concern in pediatrics. The majority of these occur in low- and middle-income countries where resources, especially subspecialty support services, are limited. Scrotal bites are relatively rare, and even fewer cases of abdominal viscus involvement have been described. No case has previously been reported of a dog bite to the scrotum leading to abdominal viscus perforation.

**Case presentation:**

A 2-year old boy presented with an acute abdomen as the result of a dog bite to his scrotum in the presence of an unrepaired inguinal hernia. Without revisiting a detailed trauma history and exam, this would have been missed, as the dog bite occurred several days prior to presentation and was nearly completely healed. The patient initially had an emergent laparotomy, small bowel resection, and hernia repair. He then suffered from a delayed anastomotic leak requiring repeat laparotomy with creation of an ileostomy. Following a prolonged post-operative course, the patient was discharged home with his ileostomy in place. He returned 3 months later to have his ileostomy reversed and was discharged after an uncomplicated operation in good condition.

**Conclusions:**

This case demonstrates the primacy of an accurate history and physical, specifically with regards to recent trauma, in the presentation of a pediatric patient with an acute abdomen. Acquiring this may involve multiple re-interviews with the family as new facts may come to light. This is especially important in resource limited areas where advanced imaging and laboratory services are not available.

## Introduction

Injuries due to dog bites are a major public health concern globally. Multiple infections including rabies, *Capnocytophagia*, *Pasteurella, methicillin-resistant Staphylococcus aureus*, and tetanus can be transmitted during dog bites [1]. This often occurs in conjunction with mechanical destruction or penetrating trauma to the affected area.

The incidence of dog bites and associated outcomes vary significantly between high-income and low- and middle-income countries (LMICs). Approximately 1–1.5% of individuals experience dog bites annually in high-income countries [1–3]. In LMICs, dog bite injuries more commonly affect children, and are associated with increased morbidity and mortality [4]. Rare cases of scrotal dog bites in adults and children have been reported previously with associated urologic trauma [5–7]. Visceral injuries related to dog bites are rare and previously reported cases have all been associated with bite wounds to the abdomen [8–12].

## Case presentation

A 2-year old, otherwise healthy, Khmu boy presented to the emergency department of a pediatric referral hospital in North-Central Laos with a two-day history of progressively worsening abdominal pain, hematemesis, and distension. This was associated with fevers, dark stools, and poor oral intake. The patient had been born at home and had no prior contacts with healthcare.

On physical examination, the child alternated between irritability and listlessness. He appeared moderately dehydrated, with associated tachycardia and fever to 39.2 °C. His abdomen was distended, rigid, diffusely tender, with percussion tenderness and absent bowel sounds. The patient had bounding peripheral pulses with flash capillary refill. His left hemiscrotum was notably enlarged with a reducible inguinal hernia.

On further investigation of the patient’s history, his mother reported that he had inguinal swelling since birth. After a delay, while re-assessing recent trauma history, the family finally reported a history of dog bite to the scrotum by a stray 2 days prior to the development of symptoms. On closer examination, the patient’s scrotum showed two small puncture marks at opposing locations on the anterior and posterior surfaces of the left hemiscrotum. There was minimal erythema around the posterior wound, but not the anterior. Neither wound demonstrated purulence, induration, tenderness, or other signs of infection. The child had suffered no other injuries during his encounter with the dog and the parents had cleaned the skin wounds at home.

**Fig. 1:**
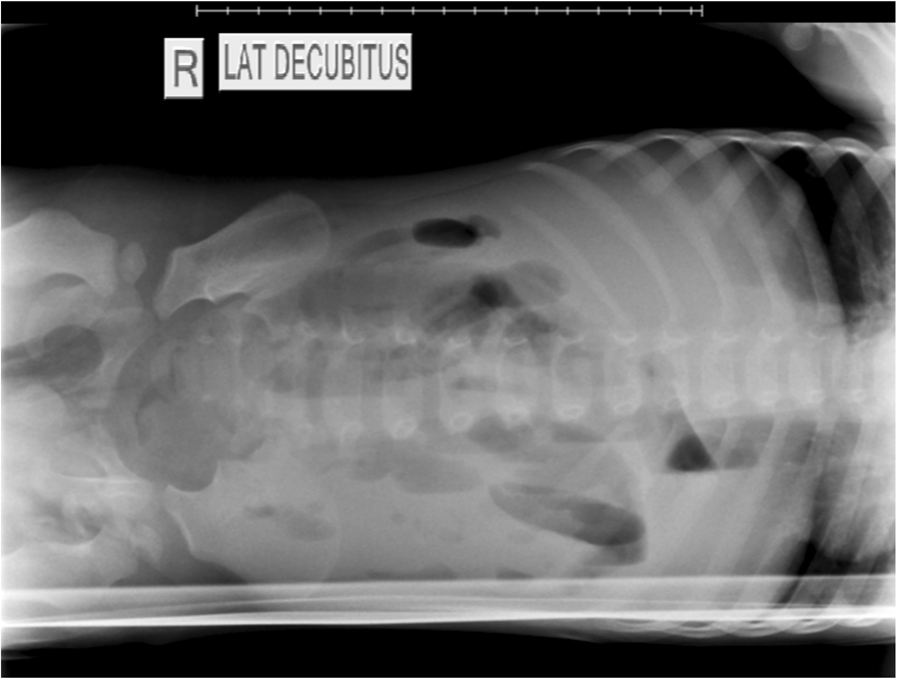
Lateral decubitus abdominal radiograph showing air-fluids levels with a sentinel loop of dilated small bowel

Initial studies revealed leukopenia (white blood cell count 3.3 × 10^9 cells/L), anemia (hemoglobin 11.3 g/dL) and uremia (blood urea nitrogen 63 mg/dL). The patient’s electrolytes were within normal limits, and a rapid typhoid test was negative. A lateral decubitus abdominal X-ray demonstrated air fluid levels with a sentinel loop of dilated small bowel (Fig. 1). He was fluid resuscitated with normal saline, then given broad-spectrum antibiotic coverage with ceftriaxone and lincomycin. He also received tetanus toxoid, and rabies vaccine due to the unknown vaccination status of the canine. Despite this resuscitation, the patient became progressively more tachycardic, and his mental status declined into obtundation. Given the patient’s acute abdomen and deteriorating status, he was taken urgently to the operating theatre.

**Fig. 2:**
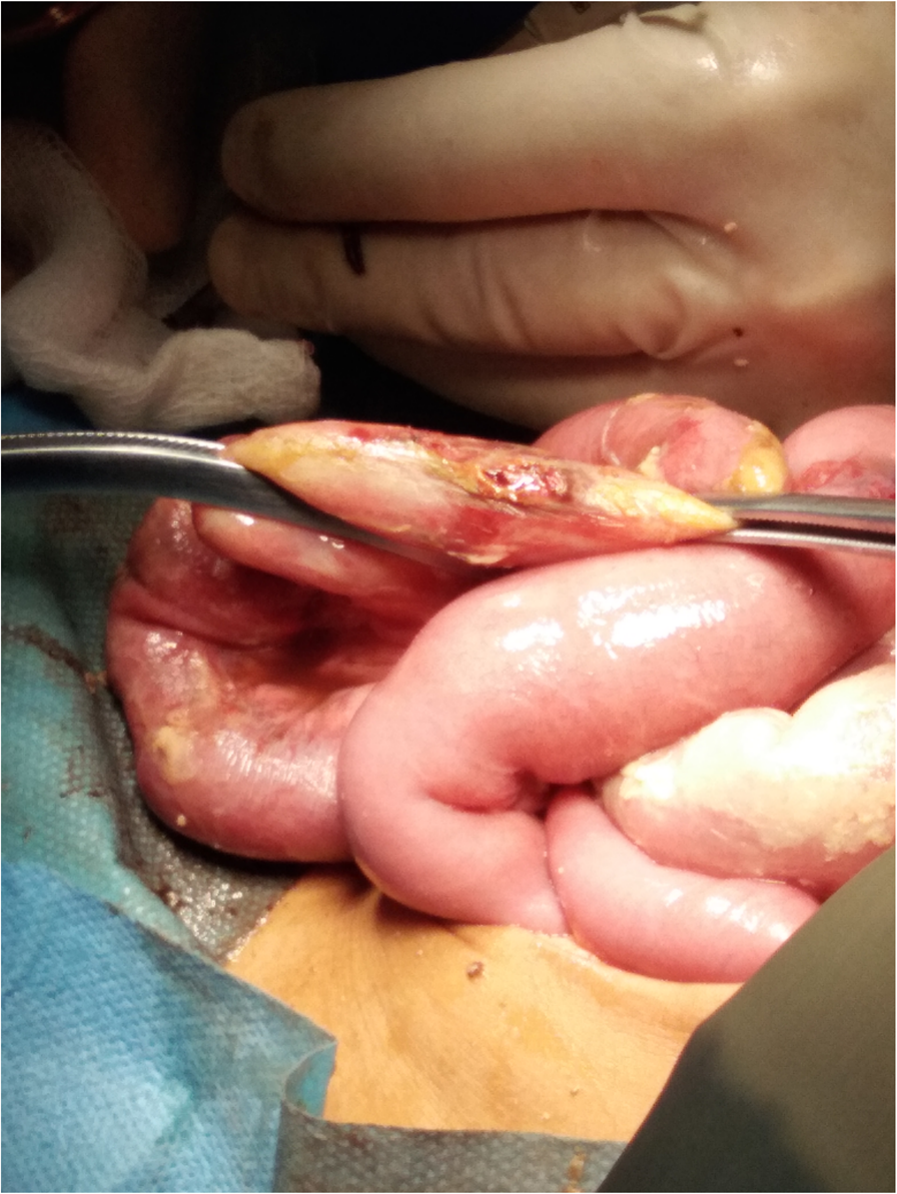
Intraoperative photograph from first operation showing perforated small bowel

During open laparotomy, the patient was noted to have purulent material throughout his abdominal cavity. Small bowel contents were herniated into the scrotum, and were manually reduced. His small bowel had two puncture perforations (Fig. 2). He ultimately had 2 cm of small bowel resected, and the inguinal hernia was repaired. On post-operative multidisciplinary review with the surgical and medical teams, the patient’s mechanism of injury was identified as a penetrating puncture into the herniated small bowel contents in his scrotum. The tract traveled superiorly to the testes and spared the spermatic cord.

The patient’s clinical course was complicated by the development of a delayed anastomotic leak requiring repeat laparotomy on post-operative day 14 with placement of an ileostomy. He had a prolonged inpatient stay following his second operation, complicated by extensive time nil per os due to concern for ongoing anastomotic leak, severe weight loss, and high-output ostomy losses. He was switched to hydrolyzed formula with slow reintroduction of F100 with improvement in his diarrhea and subsequent weight gain. Neither of his testicles were compromised in the course of his injury, or subsequent surgeries. He was discharged home after 54 days in the hospital. He returned 3 months later and his ileostomy was reversed during an uncomplicated surgery and he was ultimately discharged home in good condition.

## Discussion

Pediatric dog bite injuries are common, representing 76–94% of all animal bite injuries in LMICs [4]. Severity ranges from superficial to fatal injuries [13, 14]. Studies have shown that the rate of dog bite injuries are 3.2-fold higher in children compared to adults due to the inability to adequately defend themselves and to recognize signs of threat [15].

Scrotal injuries range significantly in severity. The most common injury involves superficial structures, with some cases developing hematomas requiring surgical management [6]. More severe cases are rare: a bite with tunica albuginea puncture and loss of testicle viability leading to orchiectomy has been described [7], with the most severe case in the literature being a total emasculation with glans, scrotal, and testicular tissue removed [16].

Penetrating visceral injuries are even more rare. To date, only five reports have described intra-abdominal injury due to dog bite trauma, and all reported cases have involved bite trauma directly to the abdomen. Three reports detail gastric perforation, with one detailing peritoneal penetration, and one case of evisceration by stray dogs with involvement of the ileum and colon [8–12].

## Conclusion

No cases in the literature have described a scrotal dog bite leading to abdominal viscus perforation. In LMICs, common surgical conditions often go unrepaired, which can lead to advanced, atypical presentations of disease. The diagnosis in this case hinged on fundamental medical craft. We demonstrate with this case the importance of thorough and systematic history taking, and the importance of revisiting this process with the family, who may have clinically relevant memories surface only on reexamination. This is especially important in low resource settings where advanced medical imaging and diagnostics are not available.

## Data Availability

All the data analyzed in this case report are contained within the published manuscript.

## Abbreviations

*LMIC*: Low- and middle-income country
